# COVID-19 control strategies and intervention effects in resource limited settings: A modeling study

**DOI:** 10.1371/journal.pone.0252570

**Published:** 2021-06-02

**Authors:** Kiran Raj Pandey, Anup Subedee, Bishesh Khanal, Bhagawan Koirala

**Affiliations:** 1 Hospital for Advanced Medicine and Surgery, Kathmandu, Nepal; 2 Nepal Applied Mathematics and Informatics Institute for Research (NAAMII), Kathmandu, Nepal; 3 Institute of Medicine, Tribhuvan University, Kathmandu, Nepal; Xavier University, UNITED STATES

## Abstract

**Introduction:**

Many countries with weaker health systems are struggling to put together a coherent strategy against the COVID-19 epidemic. We explored COVID-19 control strategies that could offer the greatest benefit in resource limited settings.

**Methods:**

Using an age-structured SEIR model, we explored the effects of COVID-19 control interventions–a lockdown, physical distancing measures, and active case finding (testing and isolation, contact tracing and quarantine)–implemented individually and in combination to control a hypothetical COVID-19 epidemic in Kathmandu (population 2.6 million), Nepal.

**Results:**

A month-long lockdown will delay peak demand for hospital beds by 36 days, as compared to a base scenario of no intervention (peak demand at 108 days (IQR 97-119); a 2 month long lockdown will delay it by 74 days, without any difference in annual mortality, or healthcare demand volume. Year-long physical distancing measures will reduce peak demand to 36% (IQR 23%-46%) and annual morality to 67% (IQR 48%-77%) of base scenario. Following a month long lockdown with ongoing physical distancing measures and an active case finding intervention that detects 5% of the daily infection burden could reduce projected morality and peak demand by more than 99%.

**Conclusion:**

Limited resource settings are best served by a combination of early and aggressive case finding with ongoing physical distancing measures to control the COVID-19 epidemic. A lockdown may be helpful until combination interventions can be put in place but is unlikely to reduce annual mortality or healthcare demand.

## Introduction

The COVID-19 epidemic, first reported in China in December 2019, was declared a global pandemic by the World Health Organization (WHO) in March 2020 [1]. Many countries have been struggling to put together a coherent strategy against the pandemic since its early days, even while infections and deaths have grown precipitously [2]. Countries with limited resources face greater difficulties against the pandemic, even as they grapple with limited resources and fewer intervention options [3–5].

Control strategies and interventions against the spread of COVID-19 could be centered around reducing the number of infected and susceptible individuals, and the contact between the two [6–9]. In the absence of a vaccine against the COVID-19 causing SARS-CoV2 virus, or an effective treatment, available control strategies are limited. They include ones that aim to reduce the number of infectious individuals (by identifying and isolating them) and ones that aim to reduce contact (with physical distancing measures, or a lockdown). Countries like Taiwan and Vietnam have been able to contain the epidemic by means of meticulous public health measures including aggressive testing, isolation, contact tracing and quarantine combined with border entry monitoring [10–12]. Korea has similarly been able to mitigate the epidemic by means of aggressive testing and isolation combined with physical distancing measures [13, 14]. China on the other hand has managed to suppress the epidemic by means of a strategy involving strictly enforced lockdowns, aggressive testing isolation and quarantine [15, 16].

Countries like Nepal, which were declared a high risk country early on in the pandemic by the WHO, stand to face difficult strategic choices and challenges given the limited amount of healthcare resources. On January 24, 2020 Nepal became the first country in South Asia to report a SARS-CoV2 infection–in a student who had returned from Wuhan, China. Although no new cases were reported until March 23, [17] Nepal tried to put together a response against a possible epidemic: First, as a precautionary measure the government began limiting international air travel from affected countries in February and later from the rest of the world. Land border crossings with India remained open until they were finally closed on March 24, 2020 [18].

Pre-emptive physical distancing measures were introduced in the middle of March, when schools were closed and annual exams canceled. The public was advised to avoid all non-essential events and gatherings. Long distance buses were closed on March 23, 2020 following which a nationwide lockdown was declared on March 24. In these three months about 10 000 RT-PCR based tests (3 per 10 000 people) for SARS-CoV2 were carried out in the country [17].

Even while Nepal has made attempts to put off a potential epidemic in the country, its response has been hindered by a lack of clarity on what the best strategy on stopping a potential epidemic might be. To study this, we built a mathematical model to simulate the consequences of adopting various strategies in preventing or controlling a possible COVID-19 epidemic in Kathmandu, Nepal. We first estimated the burden of an unmitigated epidemic. We then assessed the potential effects of implementing control interventions: a lockdown (total shutdown of movement), physical distancing (avoiding large gatherings, no handshake, minimizing physical proximity), or aggressive testing and contact tracing with quarantine. In addition, we explored the effect of these interventions when implemented together. We analysed potential demand for health services and the relative mortality burden in each of these scenarios.

## Methods

We used an age-structured SEIR (Susceptible, Exposed, Infectious, Recovered) model with heterogeneous mixing to estimate epidemic burden due to COVID-19 in Kathmandu [19–21]. We created additional compartments *Q*, *J*, *H*, *U*, and *D* for individuals in quarantine, isolation, hospital, Intensive Care Unit (ICU), and those who have passed away due to the disease, respectively. These compartments were further divided into 16 age-structured groups each and populated based on the age-specific population distribution of Nepal. Population mixing patterns were given by a Nepali population specific contact matrix [22].

In the model, susceptible individuals in the age-group *i* move from the susceptible compartment (*S*_*i*_) to the exposed compartment (*E*_*i*_) based on the force of infection (λ_*i*_) given by:

$$\begin{array}{c} \lambda_{i}=\sum_{j=1}^{16}\lambda_{ij}, \end{array}$$ (1)

where, λ_*ij*_ represents the age-specific force of infection when susceptible individuals in age-group *i* interact with infectious individuals in age-group *j*. λ_*ij*_ is the product of the age-specific transmission rate *β*_*ij*_ and the proportion of infectious individuals.

$$\begin{array}{c} \lambda_{ij}=\beta_{ij}*(\epsilon_{a}E_{j}+\epsilon_{a}\xi_{Q}Q_{j}+I_{j}+\xi_{j}J_{j}+\xi_{j}H_{j}+\xi_{j}U_{j})/N \end{array}$$ (2)

**Fig 1:**
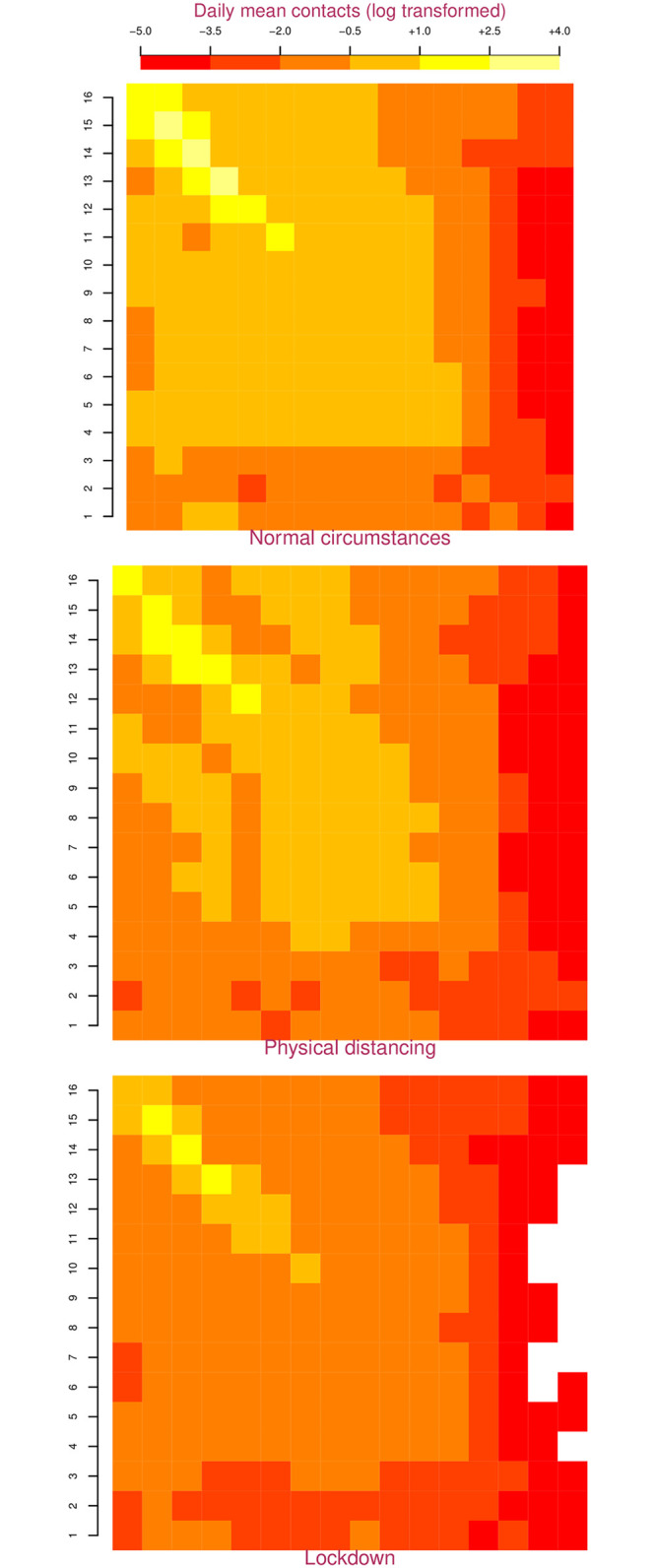
Age and intervention specific contact matrices for Nepal. In these matrices, the population is divided into 16 5-year age-groups represented along the two axes that start at the top left. The top left square represents contacts between 0–4 year olds and the bottom right square represents contacts between individuals who are 75 years and older. Colors in the squares represent the log transformed mean daily contact rate between corresponding 5-year age-groups cohorts. Dark/red colors represent fewer contacts, light/yellow colors represent a greater number of contacts. Physical distancing causes the lighter squares to darken indicating fewer contacts, while a lockdown causes the lighter squares to darken even further.

*β*_*ij*_ is the transmission rate expressed as:

$$\begin{array}{c} \beta_{ij}=m_{ij}\rho_{ij}, \end{array}$$ (3)

where, *m*_*ij*_ is the contact rate between individuals in group *i* and *j* (Fig 1), *ρ*_*ij*_ is the probability of transmission per contact between an individual in group *i* with *j*, and is modeled as a Poisson distribution as follows:

$$\begin{array}{c} \rho_{ij}=1-e^{-\eta \tau_{ij}}, \end{array}$$ (4)

*η* is the number of infections transmitted per unit time and *τ*_*ij*_ is the duration of each contact between individuals in groups *i* and *j*.

Exposed individuals move to infectious compartment (*I*) at a rate *σ* (incubation rate), where 1/*σ* is the incubation period. *ϕ*_1_ and *ϕ*_2_ represent the fraction of exposed and infectious individuals who are in quarantine (compartment *Q*) and isolation (compartment *J*) respectively. Individuals in the exposed compartment (*E*) are less infectious as compared to individuals in the infectious compartment (*I*). We account for this lower infectiousness by a factor *ϵ*_*a*_. *ξ*_*J*_ and *ξ*_*Q*_ account for the reduced infectiousness of individuals in isolation and quarantine respectively, based on the extent to which their contact with susceptible individuals is reduced [22]. For our analysis we assume that individuals sick enough to require hospitalization (compartments *H* (general ward) and *U* (ICU)) will either be isolated at the hospital, or self-isolate at home if no hospital beds are available.

We assume all deaths happen only among individuals who require hospitalization. The general ward mortality ratio (*ψ*_*i*_) is (*κ*_*i*_/*μ*_*i*_)*(1−*f*) and ICU mortality ratio, represented by *ω*_*i*_ is (*κ*_*i*_/*μ*_*i*_)*f, where *κ*_*i*_ is infection fatality ratio for the age-group *i*, *μ*_*i*_ is the proportion of individuals in age-group *i* who require hospitalization. We assume that, of all the deaths, a fraction *f* occurs among individuals who require ICU. *γ* is the recovery rate among infectious individuals that do not require hospitalization, *δ*_1_ is the recovery rate among general ward patients and *δ*_2_ is the recovery rate among patients who require ICU. Additionally, when demand for hospital beds exceeds the number of available beds the fatality ratio increases by a factor *θ* which is equal to the relative deficit of hospital beds in Kathmandu as compared to China (from where the mortality ratios are obtained). We further explain the calculation of *θ* in the S1 Appendix.

**Fig 2:**
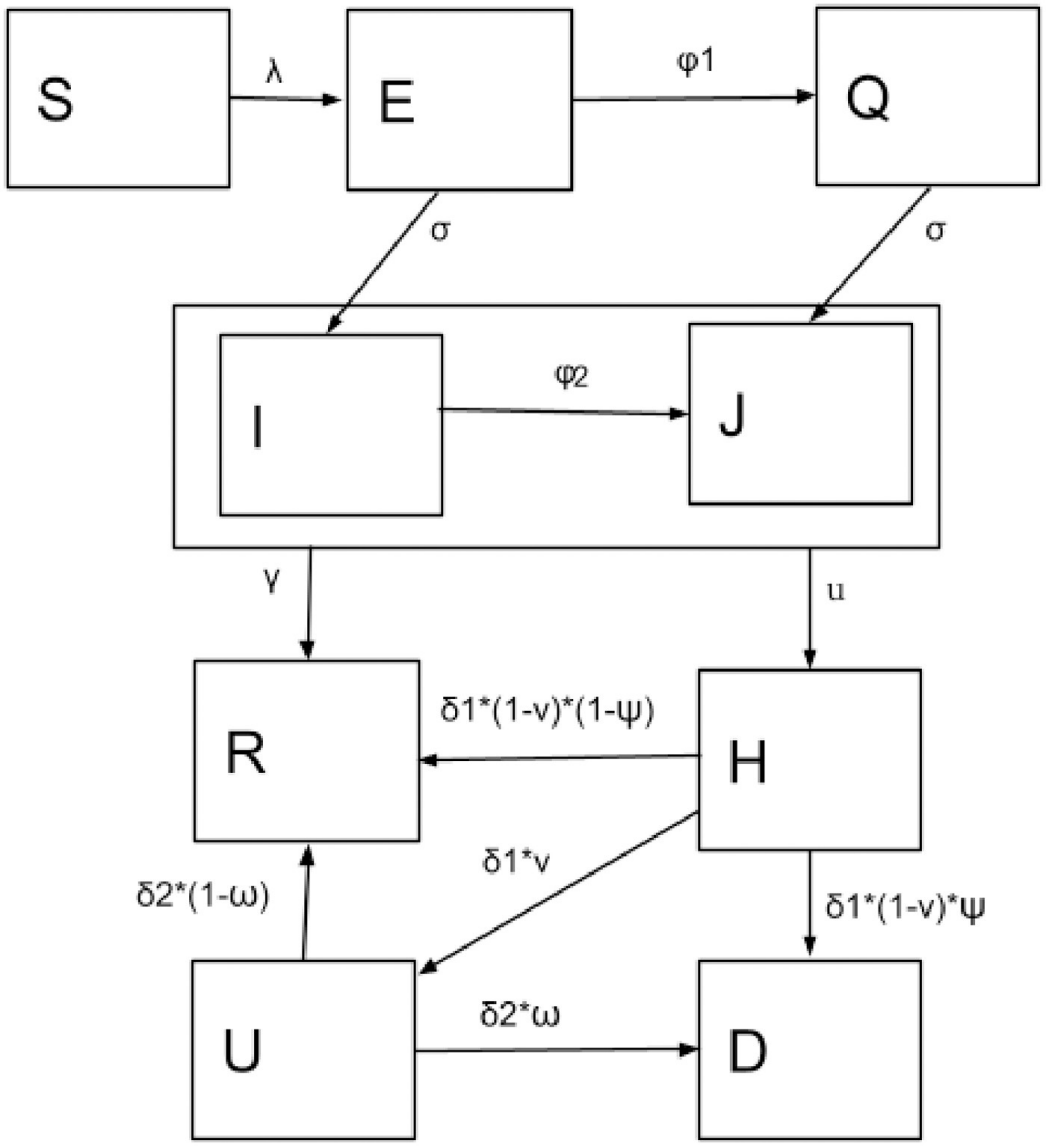
Flow diagram of the SEIR model for COVID-19 transmission dynamics. Boxes represent disease state compartments for Susceptible (*S*), Exposed (*E*), Infectious (*I*), and Removed (*R*), with additional compartments for Quarantine (*Q*), Isolation (*J*), Hospitalized in the general ward (*H*), Hospitalized in the ICU (*U*) and Dead (*D*). Arrows represent flow between compartments with the flow determined by their corresponding parameter values. Parameter values are further explained in Table 1.

Our model is described by the flow diagram in Fig 2, which corresponds to the following system of differential equations, where *i* = {1, …, 16} and represents sixteen 5-year age-cohorts.

$$\begin{array}{c} \frac{dS_{i}}{dt}=-\lambda_{i}S_{i}\left(t\right) \end{array}$$ (5)

$$\begin{array}{c} \frac{dE_{i}}{dt}=\lambda_{i}S_{i}\left(t\right)-\sigma E_{i}\left(t\right)-\phi_{1}E_{i}\left(t\right) \end{array}$$ (6)

$$\begin{array}{c} \frac{dQ_{i}}{dt}=\phi_{1}E_{i}\left(t\right)-\sigma Q_{i}\left(t\right) \end{array}$$ (7)

$$\begin{array}{c} \frac{dI_{i}}{dt}=\sigma E_{i}\left(t\right)-\phi_{2}I_{i}\left(t\right)-\mu_{i}I_{i}\left(t\right)-\gamma I_{i}\left(t\right) \end{array}$$ (8)

$$\begin{array}{c} \frac{dJ_{i}}{dt}=\phi_{2}I_{i}\left(t\right)+\sigma Q_{i}\left(t\right)-\mu_{i}J_{i}\left(t\right)-\gamma J_{i}\left(t\right) \end{array}$$ (9)

$$\begin{array}{c} \frac{dH_{i}}{dt}=\mu_{i}J_{i}\left(t\right)+\mu_{i}I_{i}\left(t\right)-\delta_{1}H_{i}\left(t\right) \end{array}$$ (10)

$$\begin{array}{c} \frac{dU_{i}}{dt}=\nu \delta_{1}H_{i}\left(t\right)-\delta_{2}U_{i}\left(t\right) \end{array}$$ (11)

$$\begin{array}{c} \frac{dR_{i}}{dt}=\gamma I_{i}\left(t\right)+\gamma J_{i}\left(t\right)+\delta_{1}\left(1-\nu \right)H_{i}\left(t\right)\left(1-\psi_{i}\right)+\delta_{2}U_{i}\left(t\right)\left(1-\omega_{i}\right)-\theta \left(\kappa_{i}/\mu_{i}\right)\left(H_{i}\left(t\right)+U_{i}\left(t\right)-\pi \right) \end{array}$$ (12)

$$\begin{array}{c} \frac{dD_{i}}{dt}=\delta_{1}\left(1-\nu \right)H_{i}\left(t\right)\psi_{i}+\delta_{2}U_{i}\left(t\right)\omega_{i}+\theta \left(\kappa_{i}/\mu_{i}\right)\left(H_{i}\left(t\right)+U_{i}\left(t\right)-\pi \right) \end{array}$$ (13)

Dead at time *t* (*D*(*t*)) also includes dead due to health service demand in excess of health system capacity.

This system of 9 equations from (5) through (13) then gives us 16 equations for each age-group resulting in a total of 144 equations for the simulation. These ordinary differential equations are solved using a solver in the deSolve package in R (v 3·6·3).

### Model parameters

The model was parameterized using published estimates of COVID-19 epidemic dynamics. We first sampled the Reproduction number (*R*_*o*_) from a uniform distribution in the interval [2,2.8] and used it to calculate the transmission rate (*β*_*ij*_) based on age-specific contact rates (*m*_*ij*_) [23, 24]. We sampled the incubation period from a uniform distribution in the interval [3, 7] days and an infectious duration of 7 days [23–25]. Transmissions per day (*η*) is calculated as the ratio of the *R*_*o*_ and the infectious duration. The average duration per contact (*τ*) is calculated as the reciprocal of the total number of contacts for an age-group. These calculations are further explained in the S1 Appendix.

We obtained age-specific hospitalization rates and mortality ratios among infected individuals from a recent analysis from China [26]. 20% of those who require hospitalization are assumed to require ICU care [27, 28]. Recovery (by getting well or death) among those who require general ward hospitalization occurs at rate *δ*_1_ and the ICU at rate *δ*_2_; *δ*_1_ and *δ*_2_ are calculated as the reciprocal of their respective lengths of stay. Infectious people who do not require hospital admission recover at a rate *γ*. All age-specific parameters were adjusted to represent the Nepali population by multiplying them with age-specific population weights [26]. Adjusting these estimates from China to the Nepali population gives an age-adjusted hospitalization rate of 2·8% (S1 Appendix).

We assumed that all patients hospitalized in the general ward require an eight day hospital stay on average; ICU stays were assumed to be six days on average. Total hospital bed capacity was estimated at 5400 (including 250 ICU beds) [28, 29]. Parameter values are given in Table 1. The parameter values are based on published estimates and current knowledge about the COVID-19 pandemic. *R*_0_ and incubation period are sampled from a uniform distribution to generate uncertainty around model estimates, given possible variability in these parameters. Transmission rate has been calculated based on a Nepal-specific contact matrix. Where appropriate, parameters have been adjusted to Nepal’s age distribution.

**Table 1:** Parameters for COVID-19 transmission dynamics in Kathmandu, Nepal.

Description	Parameter	Value (Range)	Reference
Sampled			
Reproduction number	*R*_0_	2.4[2.0, 2.8]	Uniform distribution assumed [23, 24]
Incubation period (days)	1/*σ*	3[3,7]	Uniform distribution assumed [27]
Fixed			
Infectiousness factor for individuals in the exposed compartment	*ϵ*_*a*_	0.1	Assumption [30]
Infectiousness factor for individuals in quarantine	*ξ*_*Q*_	0.3	Assumption
Infectiousness factor for individuals in isolation	*ξ*_*J*_	0.15	Assumption
Infectiousness duration (days)	1/*γ*	7	[25]
Hospitalization rate among infectious individuals % (age-specific)	*u*	2.8 (0−16.6)	[26]
Hospitalized patients admitted to ICU %	*v*	20	[31]
Infection fatality ratio % (age-specific)	*κ*	0.38 (0.0016−7.8)	[26]
Proportion of infected individuals quarantined	*ϕ*_1_	0.1	Assumption
Proportion of infected individuals isolated	*ϕ*_2_	0.1	Assumption
Average general ward length of stay (days)	1/*δ*_1_	8	Assumption [31]
Average ICU length of stay (days)	1/*δ*_2_	6	Assumption [31]
Bed capacity (General + ICU)	*π*	5400	[28, 29]
Proportion of deaths that occur in the ICU	*f*	0.8	Assumption
Dependent on other parameters			
Transmission per day (1/day)	*η*	0.34 (0.29−0.4)	Based on *R*_0_ and infectious duration*
Duration per contact (day)	*τ*	0.094 (0.031−0.301)	Based on contact rate* [22]
Transmission rate	*β*	0.0298 (0.0101−0.0927)	Based on transmissions per day and duration per contact*
General ward mortality ratio % (age-specific)	*ψ*	2.6 (0.49−9.40	Based on infection fatality ratio and hospitalization rate*
ICU mortality ratio % (age-specific)	*ω*	10.2 (1.97−37.59)	Based on infection fatality ratio and ICU admission rate*
Excess mortality factor	*θ*	{0,1.1}	If bed capacity exceeded, 1.1, else 0.*[28, 29]

Note: Parameter values are based on published estimates and current knowledge about the COVID-19 pandemic. *R*_0_ and incubation period are sampled from a uniform distribution to generate uncertainty around model estimates, given possible variability in these parameters. Transmission rate has been calculated based on a Nepal-specific contact matrix. Where appropriate, parameters have been adjusted to Nepal’s age distribution.

*Calculation of dependent parameters is further explained in the S1 Appendix.

### Interventions

**Fig 3:**
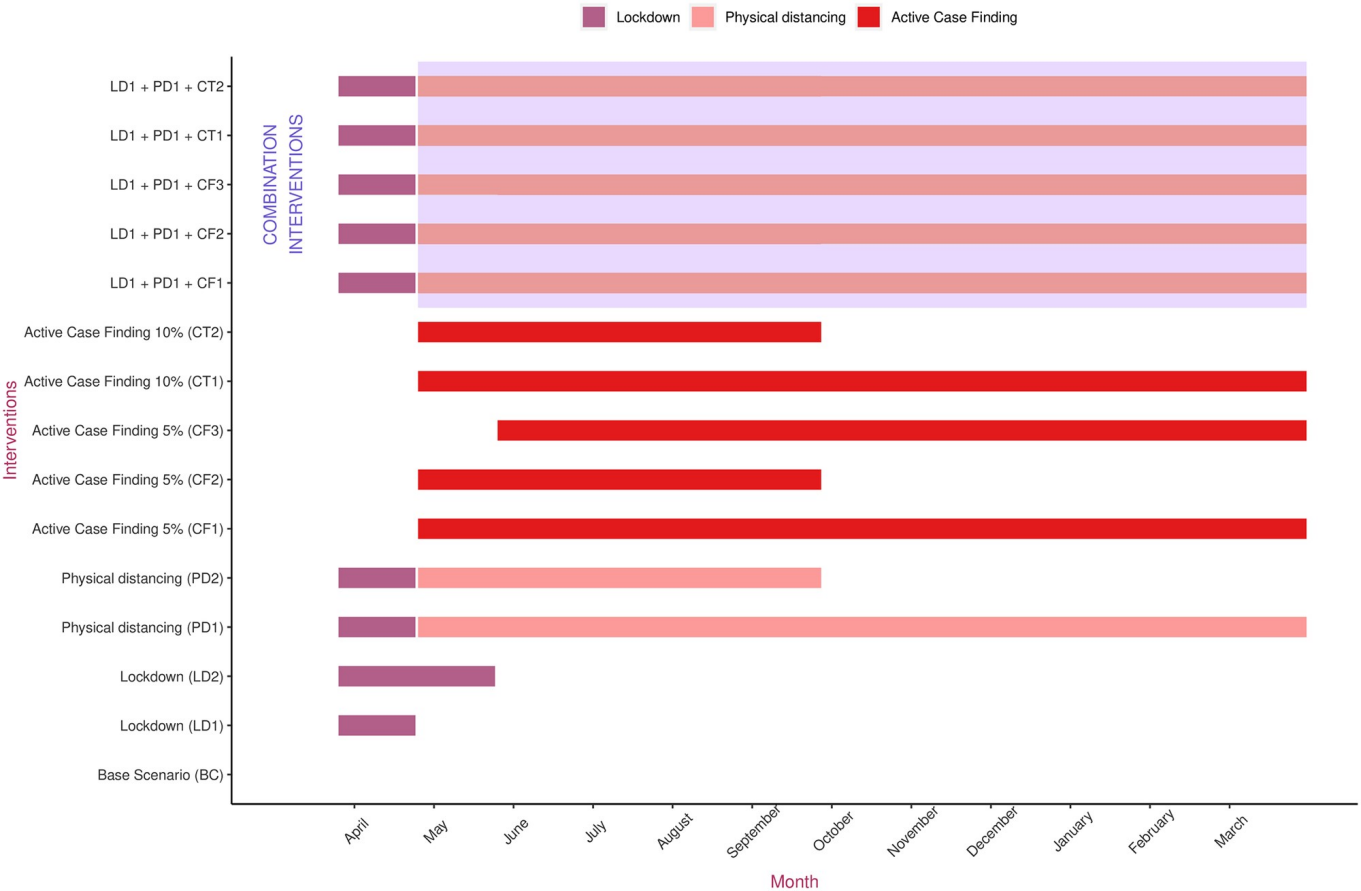
Timeline for the implementation of time-dependent interventions against COVID-19. The y-axis labels represent various interventions that were considered. The colour coded horizontal bars represent the duration the respective intervention is in place. The area on the top shaded in blue represents a combination of interventions. Interventions begin with a lockdown that began on March 24. A one year time duration beginning March 24, 2020 has been considered in this study.

A total lockdown is assumed to reduce overall contacts by 70%. Physical distancing is estimated to be only 50% as effective as lockdown in reducing contacts. Individuals placed in isolation are assumed to have contacts reduced by 75% at home and 90% across all other settings. Individuals placed in home quarantine have the same reductions but with only 50% compliance [32, 33]. These estimates are summarized in Table 2. Since a lockdown is already in place, we did not analyze the individual effect of school and workplace closures. Multi-intervention epidemic control strategies were created by a linear combination of these individual strategies. We also used time-dependent control variables to simulate the effect of time varying interventions (Fig 3).

**Table 2:** Interventions against COVID-19 and their effectiveness.

Intervention	Effectiveness	Reference
Lockdown (LD)	70% reduction in overall contacts	Assumption [34]
Physical Distancing (PD)	35% reduction in all contacts	Assumption [34]
Home Isolation	75% reduction in home contact 90% reduction in outside contact	Assumption [35, 36]
Home Quarantine	Same effectiveness as isolation but with only 50% compliance	Assumption
Active Case Finding (CF)	5% or 10% of the daily undetected infection burden detected	Assumption

Note: Assumptions are made based on the referenced citation. Active case finding assumptions are made based on probable feasibility.

We began our simulations on March 24, 2020 when the lockdown began. We assumed that there were 65 exposed and 120 infectious cases–none of which had been detected–on that day. We arrived at these numbers based on the fact that, in an average epidemiological scenario, one additional infectious case of COVID-19 on January 24–when the first case was announced–that went undetected would have led to that many cases by March 24. Our susceptible population was 2·6 million, the total population of the Kathmandu valley. For our analysis, we ignored any additional importation of infections into Kathmandu. First, we simulated a base scenario to estimate the epidemic burden and healthcare demand if no intervention had been instituted. Second, we simulated a scenario where a lockdown is in place for a month starting March 24. We also simulated a lockdown period of 2 months. Third, we simulated a scenario in which a lockdown is in place for a month, followed by physical distancing measures for the entire year, or for half the year. Fourth, we simulated a scenario where enhanced testing, isolation, contact tracing and quarantine is started on April 24, after a month-long lockdown. Enhanced testing is assumed to identify 5% of the daily burden of infections (exposed and infectious). Finally we tested a strategy combining enhanced testing, isolation, contact tracing and quarantine alongside lockdown and year-long physical distancing measures. We ran our simulations to explore the effects on potential epidemic burden and healthcare demand over a 12 month period. In the S1 Appendix, we also explore the effect of hospital capacity expansion on projected mortality burden.

### Uncertainty of model estimates

To explore the uncertainty of our model estimates, we sampled the reproduction number from a uniform distribution in the interval [2, 2.8]. The incubation period was sampled from a uniform distribution in the interval [3, 7] days. The transmission rate was calculated based on the age and location-specific contact rate. These scenarios are relevant because the burden of undiagnosed cases has been implicated as an important driver of the COVID-19 epidemic. We present the uncertainty of our model estimates in terms of their Inter-Quartile Range (IQR).

## Results

In our hypothetical base scenario of an unmitigated epidemic and no interventions, the epidemic burden is projected to peak at 100 days from March 24, 2020. Estimates of epidemic burden for this hypothetical scenario of no intervention are presented in the S1 Appendix. Demand for general ward hospital beds will peak at 108 days (IQR 97–119) from March 24 and demand for ICU beds peaking at 113 days (IQR 103–124). In this base scenario, peak demand for general ward beds is likely to exceed the current supply of 5400 beds by a factor of 9, and demand for ICU beds is likely to exceed supply by a factor of 25. These estimates do not account for healthcare demand due to non-COVID-19 illnesses.

**Fig 4:**
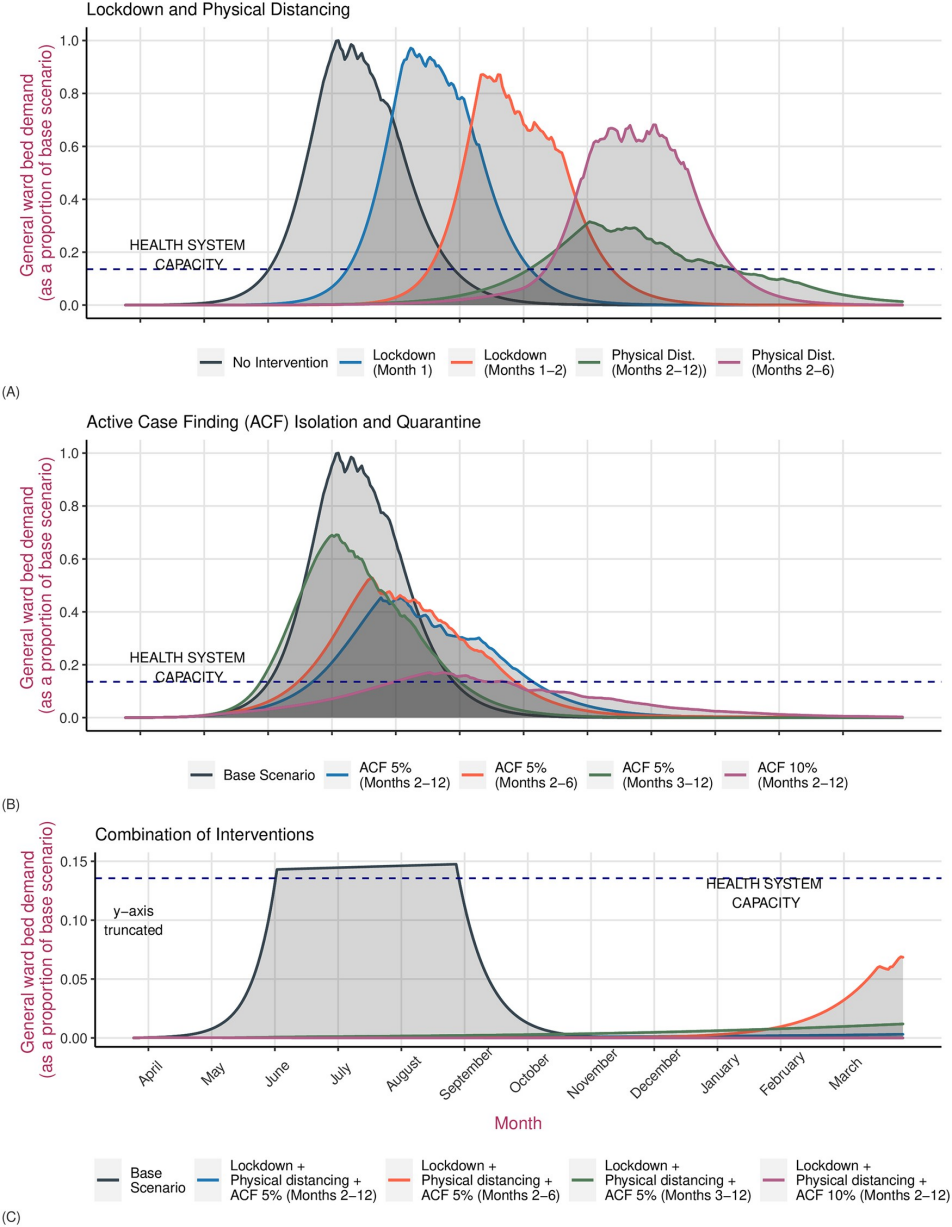
A comparison of the effectiveness of interventions against COVID-19 in reducing the demand for hospital beds. Panel (A) on the top compares lockdown or physical distancing measures implemented for a variable duration as compared to the base scenario of no intervention. Panel (B) in the middle compares active case finding measures with the base scenario. Panel (C) at the bottom compares the effectiveness of a combination of interventions with the base scenario. The blue line represents the health system capacity which is a total of 5400 hospital beds for Kathmandu, including approximately 250 ICU beds. The y-axis has been truncated in Panel (C) to accommodate observations that are closer to the x-axis.

**Fig 5:**
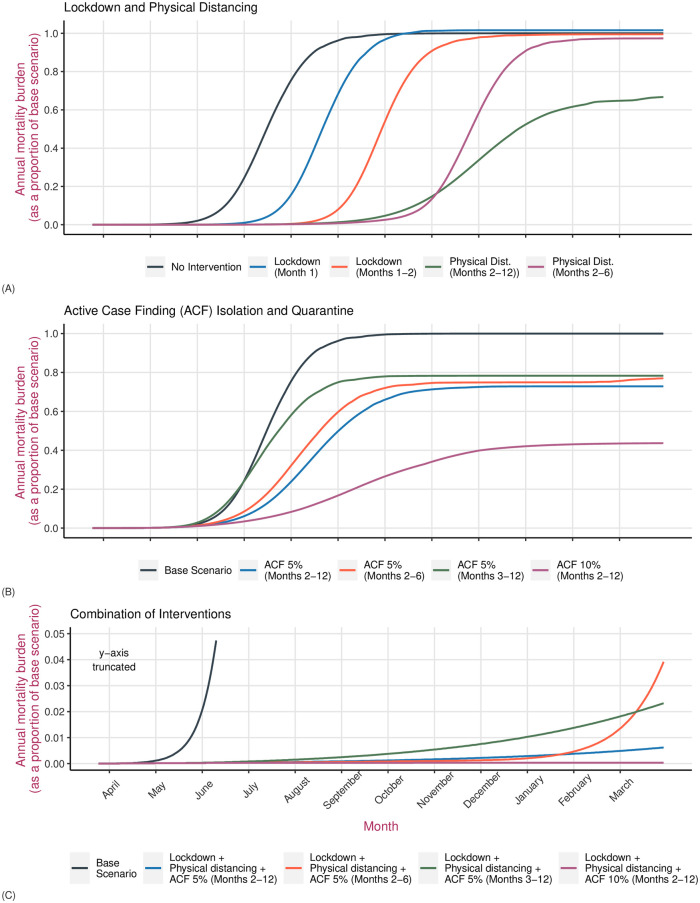
A comparison of the effectiveness of interventions against COVID-19 in reducing mortality burden. Panel (A) on the top compares lockdown or physical distancing measures implemented for a variable duration as compared to the base scenario of no intervention. Panel (B) in the middle compares active case finding measures with the base scenario. Panel (C) at the bottom compares the effectiveness of a combination of interventions with the base scenario of no intervention. The y-axis has been truncated in Panel (C) to accommodate observations that are closer to the x-axis.

A 1 month lockdown starting on March 24 will have no effect on the total number of deaths by the end of the year. Demand for healthcare will peak 36 days later (median 144 days, IQR 131–156) as compared to the base scenario, however the number of hospital admissions required will remain the same as the base scenario. A two-month lockdown will also not make any difference on the number of deaths or the number of hospital admissions required. However, healthcare demand will peak 74 days later as compared to the base scenario (Table 3). Physical distancing measures that reduce overall contact by 35%, introduced at the end of March and in place for a year, will reduce projected deaths by 33% as compared to the base scenario. Demand for healthcare will also fall significantly with peak demand, projected to occur at 245 days (IQR 216–310 days), falling by 65%. ICU demand will fall by 63% and peak a week later. Physical distancing measures that are in place just for the first six months will have a minimal impact in reducing deaths or the peak demand for healthcare, although the peak will occur more than three months later as compared to the base scenario (Figs 4 and 5).

**Table 3:** Modeled interventions against COVID-19 and their effectiveness.

Intervention	General Ward Bed Demand at Peak %*	General Ward Bed Demand Peak Day**	ICU Bed Demand Peak%*	ICU Bed Peak Demand Day**	Deaths %*
	Median IQR	Median IQR	Median IQR	Median IQR	Median IQR
Base Scenario (BC)	100.0 85.7, 112.5	107.5 97.0, 119.0	100.0 86.7, 112.0	113.0 103.0, 124.3	100.0 96.6, 103.7
Lockdown 1 Month					
Month 1 (LD1)	102.8 89.0, 115.3	143.5 131.0, 156.0	102.8 90.0, 114.4	149.5 136.8, 162.0	101.6 97.0, 104.7
Lockdown 2 Months Months 1–2 (LD2)	102.4 86.3, 115.6	182.0 166.0, 202.3	102.3 87.1, 114.9	187.5 172.0, 208.3	99.5 95.1, 104.5
Physical distancing Months 2–12 (PD1)	35.8 22.7, 45.6	245.0 215.8, 309.5	36.9 23.7, 47.1	251.0 221.0, 314.5	66.7 47.9, 77.3
Physical distancing Months 2–6 (PD2)	88.1 79.3, 92.1	239.0 218.8, 255.0	88.2 80.5, 92.9	245.0 224.0, 261.0	97.3 95.8, 100.9
Active Case Finding 5% Months 2–12 (CF1)	47.6 31.5, 61.3	139.0 117.0, 159.0	48.8 32.6, 62.5	144.5 123.0, 164.3	72.9 62.6, 81.1
Active Case Finding 5% Months 2–6 (CF2)	53.7 38.1, 64.7	130.5 115.0, 154.3	54.8 39.4, 65.8	136.0 121.0, 160.0	77.0 72.6, 82.9
Active Case Finding 5% Months 3–12 (CF3)	63.9 47.3, 81.3	105.0 93.0, 122.0	64.9 48.7, 82.1	111.0 99.0, 127.3	78.3 72.8, 86.2
Active Case Finding 10% Months 2–12 (CT1)	17.1 7.9, 25.9	170.5 146.8, 220.3	17.8 8.3, 26.9	173.0 148.8, 225.5	43.6 28.7, 54.2
Active Case Finding 10% Months 2–6 (CT2)	40.7 28.2, 63.1	280.0 139.8, 290.0	41.8 29.3, 64.2	286.0 145.0, 295.0	86.1 77.4, 90.5
LD1 + PD1 + CF1	0.3 0.0, 2.8	365.0 10.0, 366.0	0.3 0.0, 2.7	356.0 17.0, 366.0	0.6 0.1, 4.2
LD1 + PD1 + CF2	5.0 0.3, 8.0	366.0 347.3, 366.0	6.8 0.2, 47.1	365.0 349.0, 366.0	3.9 0.2, 49.5
LD1 + PD1 + CF3	1.0 0.1, 5.1	365.0 255.0, 366.0	1.0 0.1, 5.2	353.0 207.5, 366.0	2.3 0.4, 9.9
LD1 + PD1 + CT1	0.0 0.0, 0.0	10.0 9.0, 11.0	0.0 0.0, 0.0	15.0 14.0, 16.0	0.0 0.0, 0.0
LD1 + PD1 + CT2	0.1 0.0, 7.0	366.0 194.9, 366.0	0.1 0.0, 0.8	364.5 197.3, 366.0	0.1 0.1, 0.6

Note:

*Percentage as compared to Base Scenario.

**Days from the day simulation began (March 24). IQR: InterQuartile Range. Base scenario indicates an unmitigated epidemic of no interventions. In the Interventions column Month indicates the duration the specific intervention is in place. Detailed estimates are available in the S1 Appendix.

Control strategies that are focused on active case finding and isolating infected (exposed and infectious) individuals will lead to greater control of the epidemic burden and significantly reduce the demand for healthcare. If 5% of the prevalent infected people were isolated every day, following a month long lockdown from March 24, projected mortality estimates would fall by 27% from the base scenario and demand for healthcare would fall by more than 50%. Healthcare demand would peak at or after day 139 (IQR 117–159 days). If this 5% active case finding intervention were combined with physical distancing measures for the year, total projected deaths by the end of the year would fall by 99.6% with demand for healthcare showing a similar fall. However, if enhanced testing and active case finding intervention were carried out for 6 months and stopped, mortality as well as demand for healthcare increases again. Similarly, starting active case finding at day 60 instead of day 30 would result in 5% less reduction in mortality and 15% less reduction in demand for health services (Table 3).

If the active case finding intervention were to be even more enhanced, detecting 10% of cases, but in place for six months and then stopped while physical distancing measures are continued, epidemic burden, demand for healthcare and mortality would continue to remain less than one per cent of the base scenario of an unmitigated epidemic (Table 3).

## Discussion

We present a comparison of the effects of a range of possible interventions for preventing and controlling a potential COVID-19 epidemic in a resource limited setting. We find that in the base scenario of an unmitigated epidemic in Kathmandu, the demand for healthcare would significantly exceed supply by a factor of 9 for general ward beds, and by a factor of about 25 for ICU beds. Even with the ongoing lockdown, these outcomes do not change; however a lockdown can prevent an epidemic from escalating and delay its peak, providing vital time to mount other control strategies. Physical distancing interventions that are in place for an entire year would reduce deaths by about a third, and the demand for hospital beds by about two-thirds as compared to the base scenario. The effectiveness of physical distancing measures alone is significantly lower if they are in place for a shorter duration of time. Interventions that aim to actively find and isolate infected individuals are the most effective in reducing the burden of the epidemic, especially when they are combined with other interventions to reduce contact. In the absence of evidence of an uncontrolled epidemic, expansion of hospital capacity is not likely to be the most effective means of reducing potential mortality from an epidemic. Even an expansion of hospital bed capacity by a thousand beds would barely prevent a third of the excess mortality due to the deficit of health services as compared to China. S1 Appendix.

Studies and research reviews that evaluate the relative effectiveness of COVID-19 interventions are limited: when they are available they either consider only one type of intervention or have been undertaken in a different context [8, 37–40]. Therefore our study is likely to offer several important insights, especially to countries whose context matches the one we considered in this study. Our first insight is on what a lockdown, currently in place in several countries can and can not achieve. While it has bought crucial time to escalate the response against the epidemic by limiting its spread, over the longer run it cannot prevent an unmitigated epidemic if additional interventions are not immediately started. Second, no single intervention will be enough to adequately contain or mitigate the epidemic. Combining a month long lockdown with year long physical distancing measures and enhanced testing and case finding will likely limit the epidemic burden to a minimal. However such synergistic interventions need to begin early and remain in place long enough. If physical distancing as well as active case finding and contact tracing measures are started early enough but stopped in six months, this would still lead to a mortality burden and healthcare demand similar to an unmitigated epidemic. In addition, the more aggressive the early case finding interventions are, the easier long-term COVID-19 control will be.

Third, an enhanced active case finding, isolation, contact tracing intervention appears to be the cornerstone of any successful control strategy. This is understandable given recent findings that almost half of the transmission may originate from pre-symptomatic individuals [41]. Fourth, in the face of an unmitigated epidemic, health systems in resource limited settings are likely to be significantly overwhelmed, resulting in mortality rates that could be double the expected rates. This is despite accounting for the younger median age of the population. As our results demonstrate, once an unmitigated epidemic is underway, even a significant expansion of hospital bed capacity will not be enough to adequately curtail the excess mortality burden. Hence a control strategy that focuses more on the expansion of hospital capacity even when combined with lockdowns and physical distancing measures, without enhanced testing and active case finding is likely to lead to significant epidemic burden and deaths.

Our analysis using time controlled interventions allows us to evaluate the type and duration of interventions required to mount an effective control strategy. A month long lockdown and physical distancing interventions combined with an active case finding intervention instituted early is likely to effectively control a potential epidemic, however physical distancing and testing interventions have to continue for the year.

Assuming that there has been no additional importation of SARS-CoV2 infection beyond the one case we considered, given the lockdown, a very conservative estimated burden of infections in Kathmandu may be less than two hundred. Even then, assuming a 0.4–0.5% yield, daily tests would need to increase to about 2000–2500 in Kathmandu to actively find 5% of the current infection burden. Testing volumes however, remain a fraction of that. Only less than 10 000 RT-PCR based tests have been carried out in the entire country as of April 22, 2020. A similar number of antibody based rapid diagnostic tests have been carried out as well, but their accuracy has been suspect. Inaccuracy of currently available tests has made case finding difficult [42].

A control strategy that targets finding a fixed percentage of the daily burden of infections scales in a geometric fashion making it operationally challenging to implement in places with limited capacity for effective public health interventions. However, even while many countries with limited resources are finding it difficult to sufficiently escalate testing, isolation and contact tracing, some others have successfully done so [43]. Community and local government led best practices are beginning to emerge where local governments and communities are facilitating testing and quarantine of possible contacts [11, 44, 45]. Technology based solutions may additionally help in contact-tracing and following up in individuals in isolation and quarantine [46].

Given the current focus on a lockdown, we have not modeled the effect of targeted physical distancing measures like school closures or measures that target the elderly. However, these measures could be significantly important, given people in Nepal tend to live in intergenerational households that have significant contact between family members. While our use of a Nepal specific synthetic contact matrix minimises some of these concerns, the effect of targeted physical distancing measures needs to be evaluated in their local context. This could be an important area for further work.

Our analysis makes significant simplifying assumptions. First, we assume that there has been no additional importation of COVID-19 cases in Kathmandu. This is a conservative assumption, but even if there were significant additional importation of COVID-19 in Kathmandu, that scenario further strengthens our overall finding that COVID-19 epidemic burden could overwhelm health system capacity in Kathmandu. We assume that everyone is equally susceptible to the virus and that there is no immunity. However, as yet it is unclear if this assumption holds true. We make assumptions about the effectiveness of a lockdown and home isolation. We also assume that it would be possible to enforce physical distancing measures that reduce contact between individuals by about a third for the entire year, an assumption that might not hold over time as the public grows weary of such measures [47]. In addition, prolonged physical distancing measures could themselves lead to adverse health, economic and well-being outcomes–an issue that is likely to severely impact many countries that do not have adequate social safety provisions. This is another area for future work. Our use of a compartmental model offers a computationally simpler method to model the pandemic, although this approach is not likely to be methodologically valid if an epidemic is yet to be established [21, 48]. We overcame this limitation by assuming that there already were about 185 infected individuals and that local transmission was under way when we began our model simulation.

Our study offers important insights on mounting an effective response against the COVID-19 epidemic in a resource limited setting. As we have outlined above, an unmitigated COVID-19 epidemic has the potential to cause significant mortality that will be exacerbated by the unmet demand for health services. Our findings suggest that the best control strategy against the epidemic is a combination of interventions that aim to identify and isolate infected individuals and reduce contact between individuals by means of ongoing physical distancing measures. A lockdown can prevent the escalation of the epidemic, but is likely to be of limited value if no additional control measures are put in place.

## Supporting information

S1 Appendix (PDF)
