# Supplementary material for: COVID-19 control strategies and intervention effects in resource limited settings: A modeling study
Source: PLoS One. 2021 Jun 2;16(6):e0252570. doi: 10.1371/journal.pone.0252570 (PMC8172066; doi:10.1371/journal.pone.0252570)
Supplement: S1 Appendix — (PDF) [file pone.0252570.s001.pdf]

COVID-19 control strategies and intervention effects in resource limited settings: A modeling study

SA: Notes on Parameters

In this section we further explain how some of our parameters were obtained.

Contact Rates (m_{ij})

$m_{ij} = m_{ij}(h) + m_{ij}(w) + m_{ij}(s) + m_{ij}(o)$

Where (h): home, (w): work, (s): school and (o): other place of contact. We obtained values for $m_{ij}(h)$, (w), $m_{ij}(s)$, $m_{ij}(o)$ from Prem et. al.[1] Interventions that change contact patterns give a new contact matrix that reflects the change.

Duration per contact (τ):

Duration per contact (τ_i) for an age-group i is given by the reciprocal of the total number of contacts per day (m_i) for that age group.

$m_i = \sum_{j=1}^{16} m_{ij}$

$\tau_i = 1/m_i$

The duration per contact between susceptible individuals in age-group i with infectious individuals in age-group j, denoted by τ_{ij}, is the same as τ_i because for any given age-group i, all contacts across age-groups j are assumed to be of the same duration.[2] Nepal's age-distribution across all 5-year age-groups is also included in the table below:

Table S1: Age-specific total number of contacts and duration per contact				
S No.	Age group	Percentage of the population [3]	Total number of contacts (m_i) (1/day)	Duration per contact (τ_i) (day)

1	0-4 years	9.4	20.93	0.048
2	5-9 years	9.7	31.95	0.031
3	10-14 years	10.4	29.64	0.034
4	15-19 years	11.2	29.85	0.034
5	20-24 years	11.1	18.70	0.053
6	25-29 years	8.7	16.35	0.061
7	30-34 years	6.8	15.05	0.066
8	35-39 years	5.9	14.30	0.070
9	40-44 years	5.4	13.04	0.077
10	45-49 years	4.9	11.14	0.090
11	50-54 years	4.2	12.56	0.080
12	55-59 years	3.5	12.26	0.082
13	60-64 years	2.8	10.11	0.099
14	65-69 years	2.3	5.59	0.179
15	70-74 years	1.6	3.33	0.301
16	75 + years	1.8	4.88	0.205

Transmissions per day (η):

Transmissions per day (η) is simply calculated as the ratio of the Reproduction number (R_0) and the infectious duration ($1/\gamma$). Since Ro is sampled from a uniform distribution (2·0--2·8) and infectious duration is 7 days, the values of η range between 2/7 = 0.29 per day to 2.8/7 = 0.4 per day.

Transmission rate (β_{ij})

Based on the above values, the age-specific transmission rate is then calculated as follows (Equations (i) and (ii) in the main text):

$$\beta_{ij} = \rho_{ij} * m_{ij} = (1 - e^{-\eta\tau_{ij}}) * m_{ij}$$

Excess mortality factor (θ)

Calculation of the excess mortality factor is explained in Section SC:

General ward and ICU mortality ratios

The general ward and ICU mortality ratios are calculated by dividing the age-specific mortality ratios by the hospitalization rate and multiplying by the ICU death factor (f = 0.8) for ICU mortality ratio and (1-f) for the General ward mortality ratio. We assume that all deaths happen among patients who require hospitalization. Nepal's age-distribution across all 5-year age-groups is also included in the table below:

Table S2: Hospitalization rates and mortality ratios

S No.	Age group	Percentage of the population [3]	Infection fatality ratio % [4]	Hospitalization rate % [4]	General ward mortality ratio (ψ) %	ICU mortality ratio(ω)%
1	0-4 years	9.4	0.0016	0.01	3.20	12.80
2	5-9 years	9.7	0.0016	0.01	3.20	12.80
3	10-14 years	10.4	0.0069	0.0408	3.41	13.63
4	15-19 years	11.2	0.0069	0.0408	3.41	13.63

5	20-24 years	11.1	0.0309	1.04	0.59	2.38
6	25-29 years	8.7	0.0309	1.04	0.59	2.38
7	30-34 years	6.8	0.0844	3.43	0.49	1.97
8	35-39 years	5.9	0.0844	3.43	0.49	1.97
9	40-44 years	5.4	0.161	4.25	0.76	3.03
10	45-49 years	4.9	0.161	4.25	0.76	3.03
11	50-54 years	4.2	0.595	8.16	1.46	5.83
12	55-59 years	3.5	0.595	8.16	1.46	5.83
13	60-64 years	2.8	1.93	11.8	3.27	13.08
14	65-69 years	2.3	1.93	11.8	3.27	13.08
15	70-74 years	1.6	4.28	16.6	5.16	20.63
16	75 + years	1.8	7.8	16.6	9.40	37.59

SB: Estimates of Intervention Effectiveness

Here we present detailed estimates for the effectiveness of interventions that we considered in reducing the epidemic burden, health services demand and the mortality burden of the interventions, as compared to the baseline scenario. Estimates presented are the number of individuals or deaths, unless they are temporal estimates when they represent the number of days since the beginning of our simulation on March 24. For each intervention, we present the mean, median, interquartile range, maximum and minimum under each scenario. Figures in table 3 in the main text are a summary of the figures in this table, expressed as a percentage of the baseline estimates.

Table S3: Interventions against COVID-19 and their effectiveness							
Intervention	Variable	Min.	Q1	Median	Mean	Q2	Max.
Base Scenario (BC)	Peak Prevalence (Infectious)	194835	249753	291636	295356	334778	426863
Base Scenario (BC)	Infectious Peak (day)	72	91	101	102	112	140
Base Scenario (BC)	Peak Prevalence (Exposed)	133910	226911	249671	256625	290729	392857
Base Scenario (BC)	Exposed Peak (day)	67	86	96	97	107	134
Base Scenario (BC)	Gen Bed Demand at Peak	31665	39973	46397	46667	52493	64946
Base Scenario (BC)	Gen Bed Peak (day)	78	97	108	108	119	146
Base Scenario (BC)	ICU Bed Demand at Peak	4622	5772	6643	6659	7461	9017
Base Scenario (BC)	ICU Peak Day	83	103	113	114	124	152
Base Scenario (BC)	Deaths	9290	9927	10373	10277	10659	11076
Lockdown (LD1)	Peak Prevalence (Infectious)	205524	259629	299939	305182	342137	414788
Lockdown (LD1)	Infectious Peak (day)	109	125	138	138	149	178
Lockdown (LD1)	Peak Prevalence (Exposed)	136802	227313	269024	266748	307636	405029
Lockdown (LD1)	Exposed Peak (day)	104	120	132	132	144	173
Lockdown (LD1)	Gen Bed Demand at Peak	33271	41557	47701	48132	53793	63450
Lockdown (LD1)	Gen Bed Peak (day)	115	131	144	144	156	185
Lockdown (LD1)	ICU Bed Demand at Peak	4843	5991	6828	6857	7618	8836
Lockdown (LD1)	ICU Peak Day	120	137	150	150	162	191
Lockdown (LD1)	Deaths	9341	9971	10540	10371	10756	11088
Lockdown (LD2)	Peak Prevalence (Infectious)	210083	251389	299339	296906	343021	402900
Lockdown (LD2)	Infectious Peak (day)	146	160	176	180	196	219

Lockdown (LD2)	Peak Prevalence (Exposed)	129140	201857	247726	249524	295804	393189
Lockdown (LD2)	Exposed Peak (day)	140	155	170	174	190	213
Lockdown (LD2)	Gen Bed Demand at Peak	33941	40268	47521	46875	53933	62052
Lockdown (LD2)	Gen Bed Peak (day)	151	166	182	186	202	226
Lockdown (LD2)	ICU Bed Demand at Peak	4935	5799	6798	6686	7652	8680
Lockdown (LD2)	ICU Peak Day	157	172	188	192	208	231
Lockdown (LD2)	Deaths	9169	9771	10320	10241	10741	11062
Physical distancing (PD1)	Peak Prevalence (Infectious)	38933	64799	102036	100378	130331	165418
Physical distancing (PD1)	Infectious Peak (day)	180	209	239	254	303	366
Physical distancing (PD1)	Peak Prevalence (Exposed)	20709	50615	78950	83175	113651	169763
Physical distancing (PD1)	Exposed Peak (day)	174	203	233	248	297	366
Physical distancing (PD1)	Gen Bed Demand at Peak	6175	10607	16608	16327	21278	26779
Physical distancing (PD1)	Gen Bed Peak (day)	186	216	245	261	310	366
Physical distancing (PD1)	ICU Bed Demand at Peak	891	1578	2453	2408	3137	3919
Physical distancing (PD1)	ICU Peak Day	192	221	251	266	315	366
Physical distancing (PD1)	Deaths	1777	4927	6922	6322	7946	8657
Physical distancing (PD2)	Peak Prevalence (Infectious)	174154	229487	254454	249062	269278	297315
Physical distancing (PD2)	Infectious Peak (day)	172	213	233	233	249	280
Physical distancing (PD2)	Peak Prevalence (Exposed)	100078	186827	221787	220225	251415	322279
Physical distancing (PD2)	Exposed Peak (day)	166	207	228	227	243	275
Physical distancing (PD2)	Gen Bed Demand at Peak	27986	36989	40855	39947	43004	46767
Physical distancing (PD2)	Gen Bed Peak (day)	179	219	239	239	255	287
Physical distancing (PD2)	ICU Bed Demand at Peak	4080	5363	5862	5752	6185	6646
Physical distancing (PD2)	ICU Peak Day	184	224	245	244	261	293
Physical distancing (PD2)	Deaths	9310	9844	10096	10072	10367	10783

Active Case Finding 5% (CF1)	Peak Prevalence (Infectious)	32061	57011	81448	86135	110958	161562
Active Case Finding 5% (CF1)	Infectious Peak (day)	87	109	130	132	150	206
Active Case Finding 5% (CF1)	Peak Prevalence (Exposed)	41038	65514	89143	93706	119021	153095
Active Case Finding 5% (CF1)	Exposed Peak (day)	83	105	125	127	145	201
Active Case Finding 5% (CF1)	Gen Bed Demand at Peak	9280	14678	22108	22481	28599	39339
Active Case Finding 5% (CF1)	Gen Bed Peak (day)	95	117	139	141	159	215
Active Case Finding 5% (CF1)	ICU Bed Demand at Peak	1381	2170	3243	3286	4163	5649
Active Case Finding 5% (CF1)	ICU Peak Day	101	123	145	147	164	220
Active Case Finding 5% (CF1)	Deaths	5399	6436	7560	7412	8333	9083
Active Case Finding 5% (CF2)	Peak Prevalence (Infectious)	43832	64831	91890	93244	114708	166375
Active Case Finding 5% (CF2)	Infectious Peak (day)	86	107	121	130	145	257
Active Case Finding 5% (CF2)	Peak Prevalence (Exposed)	43472	74017	95195	99665	127356	163018
Active Case Finding 5% (CF2)	Exposed Peak (day)	81	102	117	123	141	250
Active Case Finding 5% (CF2)	Gen Bed Demand at Peak	10709	17777	24896	24033	30179	40212
Active Case Finding 5% (CF2)	Gen Bed Peak (day)	93	115	131	135	154	198
Active Case Finding 5% (CF2)	ICU Bed Demand at Peak	1591	2622	3640	3508	4382	5768
Active Case Finding 5% (CF2)	ICU Peak Day	99	121	136	140	160	203
Active Case Finding 5% (CF2)	Deaths	6511	7461	7988	7947	8525	9126
Active Case Finding 5% (CF3)	Peak Prevalence (Infectious)	38330	80348	112720	118689	151724	273215
Active Case Finding 5% (CF3)	Infectious Peak (day)	68	84	97	100	112	156
Active Case Finding 5% (CF3)	Peak Prevalence (Exposed)	48368	91615	126932	127024	159187	209088
Active Case Finding 5% (CF3)	Exposed Peak (day)	63	80	92	96	108	151
Active Case Finding 5% (CF3)	Gen Bed Demand at Peak	10661	22055	29633	29859	37944	55935
Active Case Finding 5% (CF3)	Gen Bed Peak (day)	77	93	105	109	122	166
Active Case Finding 5% (CF3)	ICU Bed Demand at Peak	1584	3244	4313	4317	5467	7786

Active Case Finding 5% (CF3)	ICU Peak Day	83	99	111	115	127	171
Active Case Finding 5% (CF3)	Deaths	5562	7482	8125	8041	8855	9681
Active Case Finding 10% (CT1)	Peak Prevalence (Infectious)	621	8636	19667	21457	28305	69302
Active Case Finding 10% (CT1)	Infectious Peak (day)	31	132	157	174	209	313
Active Case Finding 10% (CT1)	Peak Prevalence (Exposed)	937	10435	24477	26458	40235	61438
Active Case Finding 10% (CT1)	Exposed Peak (day)	31	129	154	171	206	312
Active Case Finding 10% (CT1)	Gen Bed Demand at Peak	223	3706	7935	8309	12081	23686
Active Case Finding 10% (CT1)	Gen Bed Peak (day)	100	147	171	186	220	326
Active Case Finding 10% (CT1)	ICU Bed Demand at Peak	33	554	1181	1231	1789	3456
Active Case Finding 10% (CT1)	ICU Peak Day	105	149	173	190	226	323
Active Case Finding 10% (CT1)	Deaths	310	2945	4527	4171	5573	7012
Active Case Finding 10% (CT2)	Peak Prevalence (Infectious)	33538	51459	84028	108989	182986	206437
Active Case Finding 10% (CT2)	Infectious Peak (day)	91	272	279	243	287	307
Active Case Finding 10% (CT2)	Peak Prevalence (Exposed)	26168	49469	70646	96797	144312	236768
Active Case Finding 10% (CT2)	Exposed Peak (day)	87	126	269	226	279	299
Active Case Finding 10% (CT2)	Gen Bed Demand at Peak	9576	13176	18906	20549	29469	33439
Active Case Finding 10% (CT2)	Gen Bed Peak (day)	100	140	280	229	290	311
Active Case Finding 10% (CT2)	ICU Bed Demand at Peak	1421	1952	2776	3006	4278	4862
Active Case Finding 10% (CT2)	ICU Peak Day	106	145	286	235	295	317
Active Case Finding 10% (CT2)	Deaths	6613	7952	8931	8591	9296	9948
LD1 + PD1 + CF1	Peak Prevalence (Infectious)	120	120	510	4293	5227	28123

LD1 + PD1 + CF1	Infectious Peak (day)	1	1	366	220	366	366
LD1 + PD1 + CF1	Peak Prevalence (Exposed)	65	65	457	4066	6727	20895
LD1 + PD1 + CF1	Exposed Peak (day)	1	1	366	238	366	366
LD1 + PD1 + CF1	Gen Bed Demand at Peak	11	13	124	1079	1303	7057
LD1 + PD1 + CF1	Gen Bed Peak (day)	8	10	365	251	366	366
LD1 + PD1 + CF1	ICU Bed Demand at Peak	1	2	18	157	183	1051
LD1 + PD1 + CF1	ICU Peak Day	6	17	356	243	366	366
LD1 + PD1 + CF1	Deaths	4	9	65	433	422	3739
LD1 + PD1 + CF2	Peak Prevalence (Infectious)	120	877	27898	61540	131137	160524
LD1 + PD1 + CF2	Infectious Peak (day)	1	336	366	341	366	366
LD1 + PD1 + CF2	Peak Prevalence (Exposed)	102	1019	29384	49046	92080	156223
LD1 + PD1 + CF2	Exposed Peak (day)	278	334	366	350	366	366
LD1 + PD1 + CF2	Gen Bed Demand at Peak	14	118	972	3693	2131	25926
LD1 + PD1 + CF2	Gen Bed Peak (day)	290	347	366	354	366	366
LD1 + PD1 + CF2	ICU Bed Demand at Peak	2	15	455	1393	3134	3792
LD1 + PD1 + CF2	ICU Peak Day	15	349	365	348	366	366
LD1 + PD1 + CF2	Deaths	6	20	407	2353	5091	8120
LD1 + PD1 + CF3	Peak Prevalence (Infectious)	120	120	1866	5071	8745	20085
LD1 + PD1 + CF3	Infectious Peak (day)	1	1	365	262	366	366
LD1 + PD1 + CF3	Peak Prevalence (Exposed)	65	148	1629	5062	9154	20007
LD1 + PD1 + CF3	Exposed Peak (day)	1	225	366	275	366	366
LD1 + PD1 + CF3	Gen Bed Demand at Peak	11	31	474	1321	2399	5260
LD1 + PD1 + CF3	Gen Bed Peak (day)	8	255	365	283	366	366
LD1 + PD1 + CF3	ICU Bed Demand at Peak	2	5	69	195	349	785
LD1 + PD1 + CF3	ICU Peak Day	14	208	353	277	366	366

LD1 + PD1 + CF3	Deaths	7	40	241	692	1020	3206
LD1 + PD1 + CT1	Peak Prevalence (Infectious)	120	120	120	120	120	120
LD1 + PD1 + CT1	Infectious Peak (day)	1	1	1	1	1	1
LD1 + PD1 + CT1	Peak Prevalence (Exposed)	65	65	65	67	67	75
LD1 + PD1 + CT1	Exposed Peak (day)	1	1	1	2	3	6
LD1 + PD1 + CT1	Gen Bed Demand at Peak	11	12	12	12	13	15
LD1 + PD1 + CT1	Gen Bed Peak (day)	8	9	10	10	11	14
LD1 + PD1 + CT1	ICU Bed Demand at Peak	1	2	2	2	2	2
LD1 + PD1 + CT1	ICU Peak Day	6	14	15	15	16	20
LD1 + PD1 + CT1	Deaths	2	3	4	4	5	15
LD1 + PD1 + CT2	Peak Prevalence (Infectious)	120	120	254	10439	5876	179013
LD1 + PD1 + CT2	Infectious Peak (day)	1	1	366	191	366	366
LD1 + PD1 + CT2	Peak Prevalence (Exposed)	65	65	293	8204	5071	94488
LD1 + PD1 + CT2	Exposed Peak (day)	1	1	366	202	366	366
LD1 + PD1 + CT2	Gen Bed Demand at Peak	11	12	33	1423	716	28709
LD1 + PD1 + CT2	Gen Bed Peak (day)	8	9	366	195	366	366
LD1 + PD1 + CT2	ICU Bed Demand at Peak	1	2	4	4	82	4118
LD1 + PD1 + CT2	ICU Peak Day	6	15	365	197	366	366
LD1 + PD1 + CT2	Deaths	2	3	7	7	60	3854
Note: Q1: First Quartile, Q3: Third Quartile. ICU: Intensive Care Unit							

SC: Effect of Hospital Capacity Expansion

The difference in access to health services is likely to result in a significant difference in mortality rates between China and Kathmandu. We accounted for this difference in the following way. Until patient volumes surpass capacity, we assume that age-specific mortality rates will remain the same as they were in China. On any day that demands exceed bed capacity, we assume that mortality rate for patients unable to find hospital beds increases proportionate to the relative deficit of per-capita hospital beds in Kathmandu as compared to China. We calculated this relative deficit by subtracting the bed ratio in Kathmandu from the ratio in China and dividing it by the bed ratio in Kathmandu. An early analysis from China has found that mortality correlates with epidemic burden beyond health service capacity.[5] We then calculated excess deaths due to the lack of healthcare. Two other factors are likely to contribute to a difference in mortality rates: age structure and comorbidities. We adjusted the mortality ratio to the first factor. Given comparable rates of risk factors like obesity and smoking between China and urban Nepal, we assume they have comparable comorbidity burden.[6–8]

With an estimated 5400 hospital beds, including about 250 critical care beds with mechanical ventilators Kathmandu has 2 hospital beds per thousand people, while China has about 4.2 beds per thousand.[9,10] Based on these figures, the excess-mortality factor (θ) is (4.2-2)/2 = 1.1, when the demand for beds exceeds capacity and 0 when the demand is below capacity. The excess-mortality factor falls to 0.7 when there are an extra thousand beds in Kathmandu and to 0.52 when there are an extra 2000 beds. In our base scenario, accounting for the relative deficit of health services in Kathmandu as compared to China (excess mortality factor) would lead to mortality to increase by 10 100 to 20 500, as compared to the base scenario. If total bed capacity were increased by a 1000, this excess mortality would fall by 32% and by 49% if capacity could be increased by 2 000 beds. If a capacity increase of 1 000 beds could be combined with a month long lockdown along with year-long physical distancing measures the excess mortality due to the deficit of healthcare would fall by 89%.

Bibliography

1. Prem K, Cook AR, Jit M. Projecting social contact matrices in 152 countries using contact surveys and demographic data. PLoS Comput Biol. 2017;13: e1005697. doi:10.1371/journal.pcbi.1005697

2. Saunders-Hastings P, Quinn Hayes B, Smith R, Krewski D. Modelling community-control strategies to protect hospital resources during an influenza pandemic in Ottawa, Canada. PLoS ONE. 2017;12: e0179315. doi:10.1371/journal.pone.0179315

3. Population of Nepal 2019 - PopulationPyramid.net [Internet]. [cited 20 Feb 2021]. Available: https://www.populationpyramid.net/nepal/2019/

4. Verity R, Okell LC, Dorigatti I, Winskill P, Whittaker C, Imai N, et al. Estimates of the severity of coronavirus disease 2019: a model-based analysis. Lancet Infect Dis. 2020;20: 669–677. doi:10.1016/S1473-3099(20)30243-7

5. Ji Y, Ma Z, Peppelenbosch MP, Pan Q. Potential association between COVID-19 mortality and health-care resource availability. Lancet Glob Health. 2020;8: e480. doi:10.1016/S2214-109X(20)30068-1

6. Rawal LB, Kanda K, Mahumud RA, Joshi D, Mehata S, Shrestha N, et al. Prevalence of underweight, overweight and obesity and their associated risk factors in Nepalese adults: Data from a Nationwide Survey, 2016. PLoS ONE. 2018;13: e0205912. doi:10.1371/journal.pone.0205912

7. Chen Y, Peng Q, Yang Y, Zheng S, Wang Y, Lu W. The prevalence and increasing trends of overweight, general obesity, and abdominal obesity among Chinese adults: a repeated cross-sectional study. BMC Public Health. 2019;19: 1293. doi:10.1186/s12889-019-7633-0

8. Ashwell M, Gunn P, Gibson S. Waist-to-height ratio is a better screening tool than waist circumference and BMI for adult cardiometabolic risk factors: systematic review and meta-analysis. Obes Rev. 2012;13: 275–286. doi:10.1111/j.1467-789X.2011.00952.x

9. Hospital beds (per 1,000 people). In: The World Bank Data [Internet]. 2020 [cited 23 Apr 2020]. Available: https://data.worldbank.org/indicator/sh.med.beds.zs

10. Paneru H. Intensive care units in the context of COVID-19 in Nepal: current status and need of the hour. JSAN. 2020;7.
